# Carcinoma Cuniculatum: A Rare Malignancy With Unique Diagnostic Dilemmas

**DOI:** 10.7759/cureus.37453

**Published:** 2023-04-11

**Authors:** Michael Nagai, Benjamin Pastwik, Alfredo Aguirre, Mark Burke, John Campbell

**Affiliations:** 1 Head and Neck Surgery, Erie County Medical Center, Buffalo, USA; 2 Emergency Medicine, Virginia Commonwealth University, Richmond, USA; 3 Oral Diagnostic Services, University at Buffalo, Buffalo, USA; 4 Head and Neck Surgical Oncology, Erie County Medical Center, Buffalo, USA; 5 Oral and Maxillofacial Surgery, Erie County Medical Center, Buffalo, USA

**Keywords:** head and neck cancer surgery, oral and maxillofacial surgery, malignancy, epithelial tumor, carcinoma cuniculatum

## Abstract

Carcinoma cuniculatum is an extremely rare and often indolent cancer that can mimic a benign process, such as osteomyelitis or odontogenic infections. This results in a definitive diagnosis that is delayed. To complicate the evaluation of this uncommon neoplasm, biopsies are often misinterpreted secondary to an incorrectly obtained tissue sample. Incisional biopsy needs to be done in a specific manner with a high degree of clinical suspicion incorporated into the patient assessment for the most accurate diagnosis. Local and distant failure rates are low with aggressive surgical resection and surgery upfront remains the treatment of choice when feasible. We present two cases that highlight the difficulty in accurate diagnosis and management of these rare cancers.

## Introduction

Carcinoma cuniculatum (CC) is an extremely rare malignant neoplasm that is seen in the oral cavity. Often misdiagnosed, these tumors can mimic osteomyelitis, dental abscess, or odontogenic cysts in their presentation, and even with multiple incisional biopsies, the definitive diagnosis can be delayed [1,2]. Currently, CC is classified by the World Health Organization (WHO) as a malignant epithelial tumor in which diagnosis on biopsy specimens can be difficult [3]. A sub-category of well-differentiated squamous cell carcinoma with unique histologic findings, it encompasses an indolent course that creates challenges with timely diagnosis and management. An important clinical characteristic, often found on biopsy, is the expression of a thick keratinaceous exudate that can be mistaken for purulence and thus, incorrectly interpreted as an infectious process. This is a critical finding and should prompt a high index of suspicion for CC. A liberal and deep biopsy is requisite as the endophytic nature of this tumor is prone to false negative biopsies on superficial sampling [4].

From an ablative standpoint, these malignancies often present with a lack of a clear clinical delineation of the tumor, making oncologic extirpation difficult. Prior surgery and delays in diagnosis can create an environment in which chronic inflammation and or tumor progression move beyond the border of what is clinically visible. Since complete surgical removal with negative margins provides excellent rates of local control it is imperative that these tumors be cautiously excised with adequate margins [5].

Local and distant failure rates are low with aggressive surgical resection and surgery upfront remains the treatment of choice when feasible [2]. We present two cases that highlight the difficulty in diagnosis and management of these rare cancers.

## Case presentation

Case 1

A 51-year-old female nurse presented to a local surgeon for management of a presumed osteomyelitis, based on history, radiographic and clinical information. She reported extraction of a left mandibular molar approximately 1.5 years prior with a slow progression of pain. Two additional teeth were subsequently removed in the months prior to presentation without resolution of her symptoms.

Past medical history was significant for osteopenia and Crohn’s disease, but otherwise unremarkable. Fosamax (alendronate) had been started around the time of initial symptom onset but had been discontinued prior to her referral. She was an everyday smoker of one pack for 35 years and consumed alcohol socially. Medications on initial evaluation were Pentasa (mesalamine), ibuprofen and a multivitamin.

**Figure 1:**
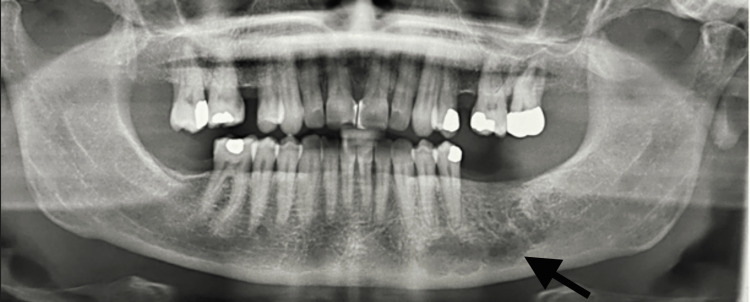
Panoramic image of patient one at the time of initial presentation showing a loculated radiolucent lesion in the body of the left mandible.

Initial exam revealed mild left mandibular swelling with no overlying skin changes. Palpation of the left mandible elicited pain. Ipsilateral lymphadenopathy was noted. There was no trismus but the mucosa over the left posterior mandible displayed ill-defined erythema, edema, or tenderness. A panoramic radiograph was significant for a lytic lesion in the left posterior mandible with intact inferior border (Figure 1). The plan was for surgical debridement under intravenous sedation. The surgery revealed both scar and granulation-type tissues along with areas of soft bone. No purulence was encountered. The surgical site was closed primarily and post-operative Ciprofloxacin 500mg q12h was initiated for a 14-day course with improvement in the patient's symptoms. Pathological analysis from the surgery revealed multiple fragments of bland, stand-alone stratified squamous epithelium with hyperkeratosis and acanthosis. Separate fragments of dense fibrous connective tissue with patchy chronic inflammation, granulation tissue with subacute inflammation, and fragments of bone were present. Some of the bone fragments were necrotic. The microscopic diagnosis was compatible with medication-related osteonecrosis of the jaw (MRONJ) and the patient had been on alendronate (a bisphosphonate class of medication), however, this had been discontinued over a year prior to her symptom onset.

**Figure 2:**
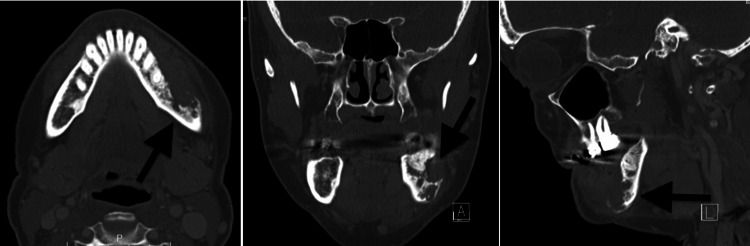
Computerized tomography for patient one showing significant osteolysis and progression of the pathologic process.

Two months following initial debridement there was an abrupt increase in pain and edema over the left face and a course of antibiotics was initiated (Ciprofloxacin 500mg q12h x 14 days) without improvement. After failing outpatient therapy, the patient was admitted to the hospital, a peripherally inserted central catheter (PICC) line was placed and infectious disease was consulted. Computed tomography at that time showed the progression of the lytic changes in the mandible, now involving the inferior border (Figure 2). Rocephin (Ceftriaxone) via PICC and Flagyl (Metronidazole) orally were started with a planned prolonged course. Additional surgical debridement was performed and thick semi-solid, cheese-like keratinaceous material resembling toothpaste was encountered. Microscopic examination revealed multiple fragments of stratified squamous epithelium, some displaying dysmaturation, hyperkeratosis, and keratin pearl formation. Granulation tissue and fragments of necrotic bone were also present. A descriptive diagnosis was rendered: Bone sequestra, fragments of epithelium with focal keratinocyte atypia, hyperkeratosis, granulation tissue, and fibrous connective tissue with chronic inflammation. The unusual clinical and microscopic presentation of this case prompted external consultation with additional oral and maxillofacial pathologists. The consultant opinions ranged from; MRONJ/possible inflamed odontogenic keratocyst (OKC), OKC with early developing verrucous carcinoma, possible squamous cell carcinoma arising in a cyst/primary intraosseous odontogenic carcinoma, and carcinoma cuniculatum. The consensus was that a liberal, deep biopsy, rather than curettage was needed to arrive at a definitive diagnosis.

**Figure 3:**
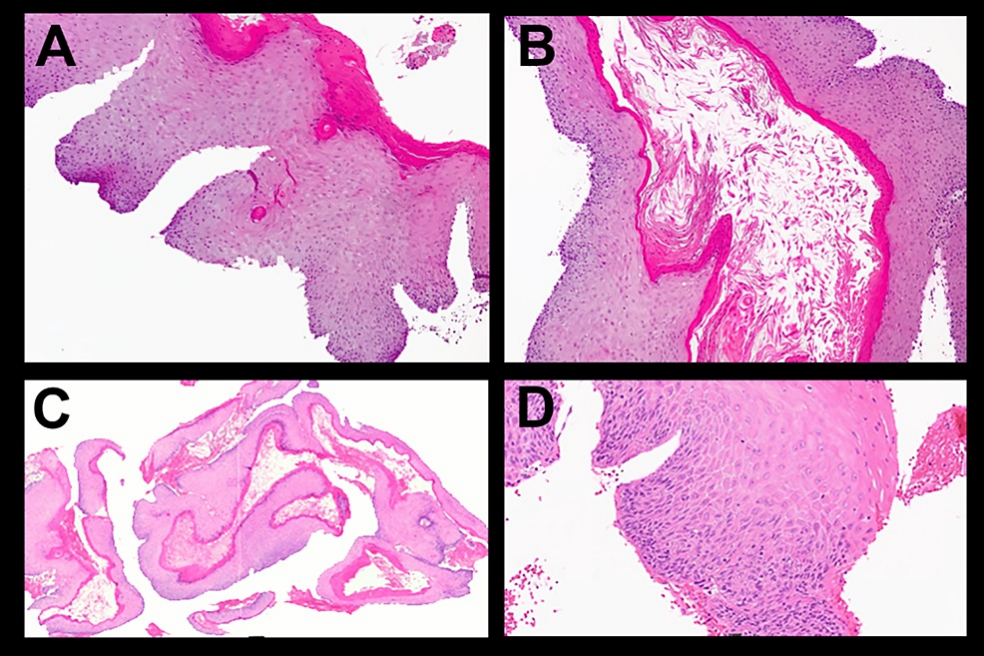
Histologic images for patient one Tissue specimens stained with Hematoxylin and Eosin (H&E) stain showing fragments of stratified squamous epithelium with verruca-papillary and endophytic architecture, hyperkeratosis, and minimal dysplastic changes confined to the basal and lower spinous cell layers. Keratin-filled burrows are also present. Final histologic diagnosis was carcinoma cuniculatum.

At five months, following multiple rounds of surgical debridement and extended intravenous antibiotic therapy, the patient’s symptoms had not resolved. She was again taken to the operating room for wide exposure and debridement. Significant tissue specimens were collected from multiple regions and submitted again for histologic evaluation. Multiple tissue sections showed fragments of stratified squamous epithelium with verruca-papillary and endophytic architecture, hyperkeratosis, and minimal dysplastic changes confined to the basal and lower spinous cell layers. Typical burrows filled with keratin and occasionally necrotic bone fragments were identified. The epithelial fragments were devoid of fibrous connective tissue. Final histologic diagnosis was carcinoma cuniculatum (Figure 3). Virtual surgical planning was utilized to facilitate the definitive surgical plan which included composite segmental resection of the left hemi-mandible along with ipsilateral selective neck dissection and reconstruction with a vascularized fibula flap. The patient underwent the procedure and recovered well without incident. The final pathologic specimen revealed CC with osseous invasion, negative margins, no perineural/lymphovascular invasion (PNI/LVI) and no pathologic nodes (pT4N0). The patient did not undergo any adjuvant therapy and is currently well, at sixteen months from surgery, with no evidence of disease (NED).

Case 2

**Figure 4:**
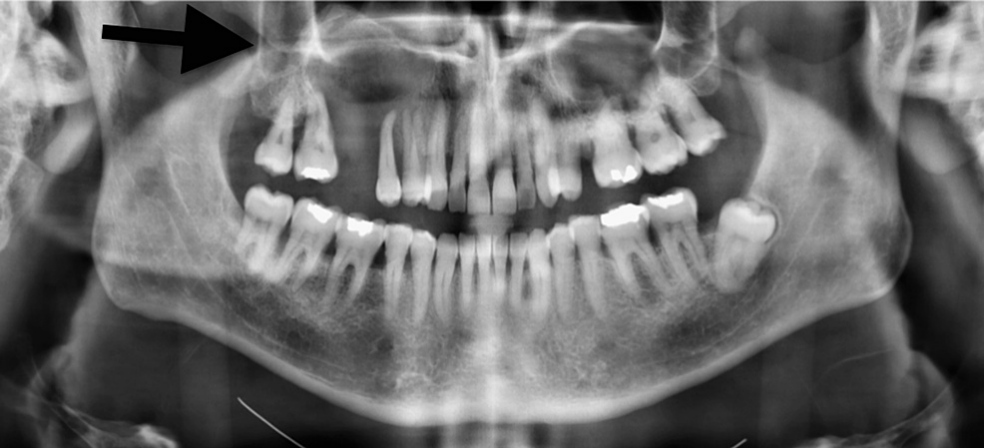
Panoramic image of Patient Two at the time of initial presentation showing an ill-defined, expansile and radiolucent lesion encompassing the right maxilla and sinus.

A 51-year-old male was seen by his general dental provider for a suspected odontogenic infection in the right maxilla. Past medical history was significant for a 10-pack-year smoking history, however, the patient had quit approximately 15 years prior. He was not on any medications. Symptoms included an expanding lesion around the gingiva/palatal region of teeth #2-5 that was thought to be fluctuant and likely related to an underlying abscess vs odontogenic cyst. Panorex from the referring dental provider shows an ill-defined, expansile, and radiolucent lesion encompassing multiple teeth in the right maxilla (Figure 4).

**Figure 5:**
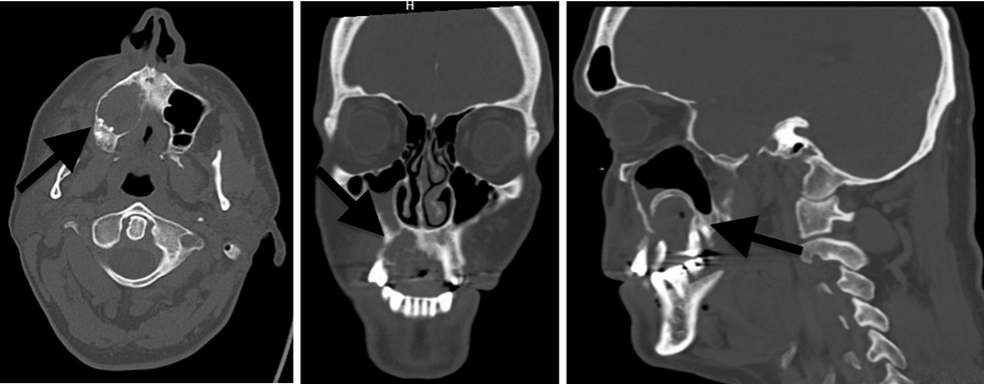
Computerized tomography for patient two showing an expansile and lytic lesion of the right maxilla.

**Figure 6:**
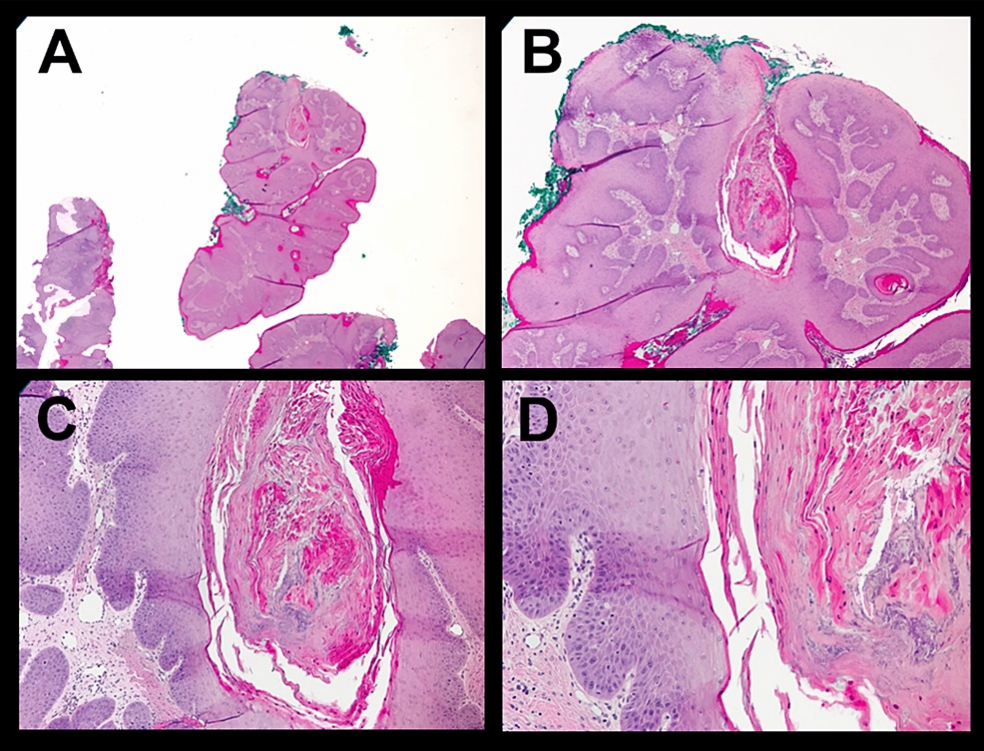
Histologic images for Patient Two Tissue specimens stained with Hematoxylin and Eosin (H&E) revealed both superficial papillary and endophytic architecture of the epithelium forming tunnels into the connective tissue with branching crypts filled with keratin. Minimal keratinocyte atypia and occasional aberrant mitotic figures were present. The final histologic diagnosis was Carcinoma Cuniculatum.

Tooth #3 was extracted in a routine fashion shortly after the initial exam with what was described as thick purulent material expressed during the procedure. The patient was placed on a course of oral antibiotic therapy without resolution of the lesion or symptoms (similar to case 1). The patient returned to his general dental provider within a month of the procedure with increasing swelling of the right maxilla and palate, now with luxation of adjacent teeth. The dentist subsequently referred the patient to a local oral surgeon who was concerned for an aggressive process secondary to his history and clinical presentation. Imaging confirmed a destructive ill-defined lesion encompassing the right maxilla with significant expansion (Figure 5). The surgeon performed a deep incisional biopsy in the clinic where multiple specimens were taken from both the gingival and palatal regions. Microscopic examination revealed both superficial papillary and endophytic architecture of the epithelium forming tunnels into the connective tissue with branching crypts filled with keratin and occasional bacterial colonies. Only minimal keratinocyte atypia and occasional aberrant mitotic figures were present. Stromal eosinophilia was also significant. The final histologic diagnosis was carcinoma cuniculatum (Figure 6).

Virtual surgical planning was utilized and the patient subsequently underwent a right maxillectomy, selective neck dissection, and reconstruction with a vascularized fibula-free flap. The patient underwent the planned procedure and has recovered well. The final pathologic specimen revealed CC with osseous invasion, negative margins, no perineural/lymphovascular invasion (PNI/LVI), and no pathologic lymph nodes (pT4N0). The patient did not undergo any adjuvant therapy and is currently well, at thirteen months from surgery, with no evidence of disease (NED).

## Discussion

Initially described in a case series by Arid et al. in 1954 as a low-grade plantar neoplasm, CC has been described at various anatomic sites throughout the body [6]. Oral CC was first described by Flieger and Owiński [7] and has remained a rarity throughout scientific journals with only a handful of case reports and small series published in the English literature [8]. Estimated at an incidence rate of <1% compared to other histologic variants of squamous cell carcinoma [9], controversy still remains if CC should be a separate entity or is simply a variant of verrucous carcinoma. Verrucous carcinoma and CC have been used synonymously in the past and CC has even been described as an inverted verrucous carcinoma [10]. However, defining histologic and clinical characteristics put this cancer into its own category with excellent prognosis and good long-term survival rates [2].

The diagnostic dilemma stems from the indolent course of the disease, inadequate harvesting of tissue for microscopic examination, and lack of awareness of both pathologist and surgeon on the clinical and morphologic characteristics of CC [11]. Both cases demonstrate the difficulties that CC presents with regard to the clinical and histologic findings. The clinical course is slow, without the classic worrisome findings often associated with malignant disease. Many times there are limited or no mucosal findings and if bony destruction is found it is often subtle and - as presented - can appear as an infectious or inflammatory condition. Definitive treatment remains aggressive surgical excision without the absolute need for regional lymphadenectomy in a clinically N0 neck [5]. In both our cases, reconstruction of the ablative defect was done with vascularized tissue transfer and so elective selective neck dissection was performed simultaneously as the neck access was already planned. From an ablative standpoint, this entity creates significant challenges with proper identification of the surgical margin. Both patients had previously undergone multiple surgeries and in the first case, three rounds of debridement. There was no clinical evidence of tumor and the overlying gingiva of the mandible was normal in appearance. Also, there was no obvious border of bone destruction, more of a zone of ill-defined radiolucency, again posing a challenge as to defining the resection margin. Virtual surgical planning was employed in both cases to allow for additional decision-making capacity of the resection/reconstruction prior to definitive surgery.

During surgical manipulation, i.e., drainage or debridement, these lesions may expel yellowish foul-smelling secretions or a thick keratinaceous debris similar in consistency to toothpaste. To prevent a delay in diagnosis it is recommended that a biopsy of the lesion be deep, as the characteristic infiltrative growth pattern of CC may not be evident at superficial levels [12-14]. Following this, definitive management with surgical resection as governed by the National Comprehensive Cancer Network (NCCN) [5] guidelines is recommended.

## Conclusions

It is imperative that providers who encounter these potential patients have a high degree of suspicion and critically evaluate the cases not behaving in a typical manner. Correlation between the clinical, pathological, and imaging findings often needs to be evaluated together to arrive at the diagnosis. CC is a distinct clinical entity and should be considered anytime an unexplained osteomyelitis or a verrucous growth of long-standing duration is encountered. The very nature of CC is that it mimics other clinical entities. Even the most experienced clinician must remain vigilant when evaluating these lesions. Subtle clues can avoid disease progression and increasing patient morbidity. Once identified, surgical resection with the standard oncologic approach followed by close surveillance should result in excellent disease control and survival rates.
